# Supplementary material for: Melanocortin 3 receptor regulates hepatic autophagy and systemic adiposity
Source: Nat Commun. 2025 Feb 16;16:1690. doi: 10.1038/s41467-025-56936-1 (PMC11830824; doi:10.1038/s41467-025-56936-1)

Supplementary Information

Melanocortin 3 receptor regulates hepatic autophagy and systemic adiposity

Patel TP., Jun JY. et al,

Supplementary Fig 1.

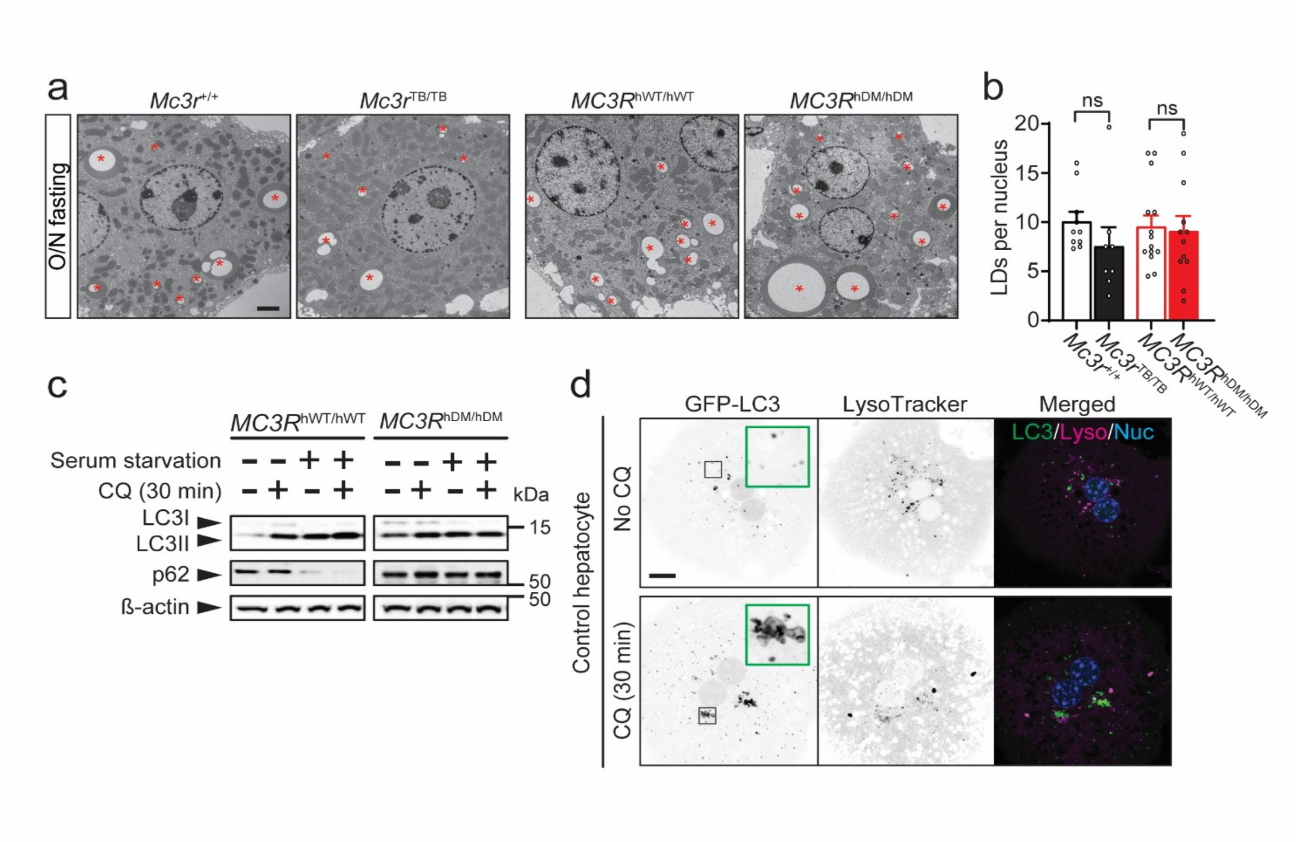

Supplementary Fig 1. a Liver transmission electron microscope images of lipid droplets (LDs), marked by asterisks, from overnight (O/N) fasted *Mc3r*$^{+/+}$, *Mc3r*$^{TB/TB}$, *Mc3r*$^{hWT/hWT,}$ *and MC3R*$^{hDM/hDM}$ mice. **b** LD numbers were quantified from TEM images (*n*=8-14 images/genotype from two independent experiments) in (a). **c** Representative western blot images of primary hepatocytes from *Mc3r*$^{hWT/hWT}$ and *MC3R*$^{hDM/hDM}$ mice are shown. The primary hepatocytes were treated with 10mM chloroquine (CQ) treatment with/without serum starvation for 1 hour. LC3I, LC3II and p62 were blotted. **d** Representative fluorescent images of *Mc3r*$^{+/+}$ transgenic mice carrying GFP-LC3 are shown after staining with Lysotracker Red and DAPI with/without 10 mM CQ treatment. The LysoTracker and Merged panels are magnifications of the region indicated by the green box in the leftmost panels. Asterisks indicate LD. Data are represented as mean±SEM. Groups were compared by unpaired two-tailed Student's t test (**b**). Scale bar, 2 µm (**a**) and 10 µm (**d**).

Supplementary Fig 2.

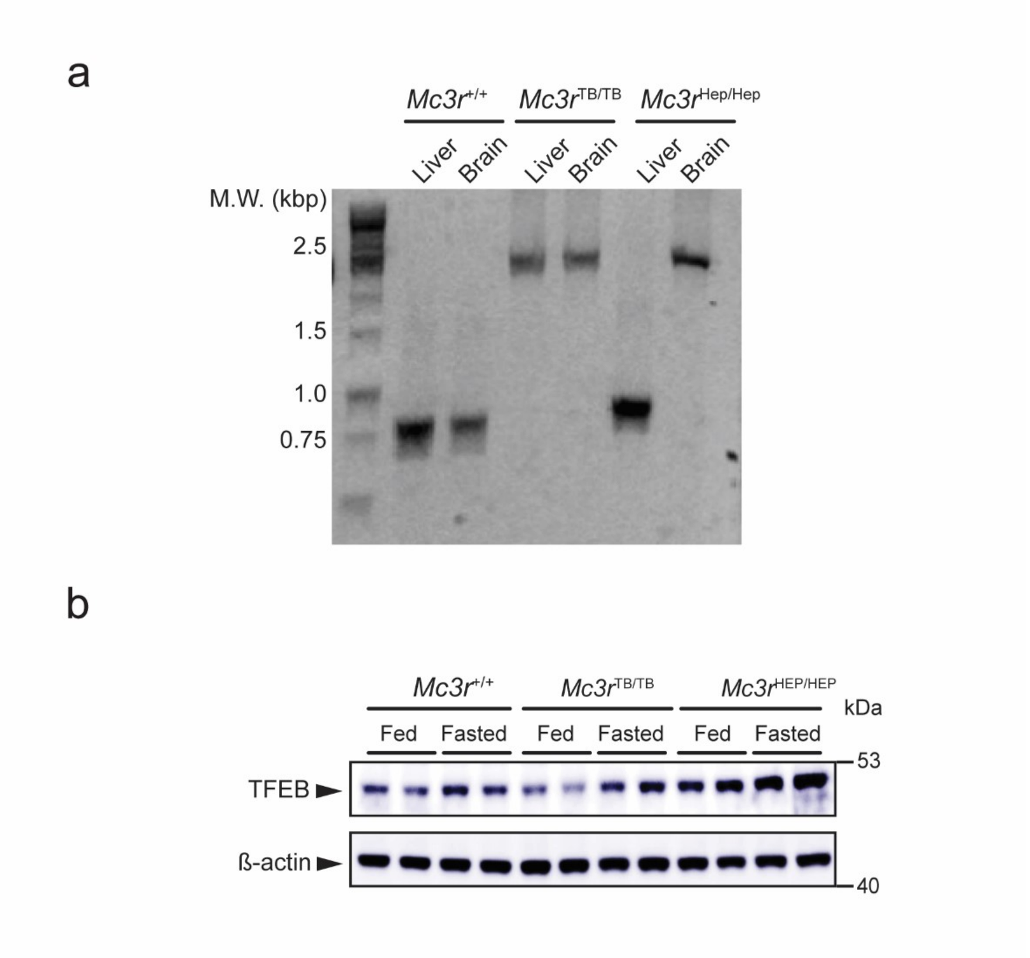

Supplementary Fig. 2. a Excision of the floxed stop sequence (or transcriptional blocker (TB)) in mice with *Mc3r*$^{TB/TB}$ who were crossed with mice expressing Cre recombinase under the control of the Albumin promoter (Alb-Cre) to produce mice homozygous for both Alb-Cre and *Mc3r*$^{TB/TB}$ (*Mc3r*$^{Hep/Hep}$) is confirmed on gel electrophoresis. Liver and brain DNA were extracted from *Mc3r*$^{+/+}$, *Mc3r*$^{TB/TB}$, and *Mc3r*^{HepHep} mice and subjected to PCR amplification using primers that surround the site subject to transcriptional blockage of *Mc3r*$^{TB/TB}$. TB insertion is 2614bp in size from MC3R$^{TB/TB}$, and only liver (but not brain) from *Mc3r*$^{Hep/Hep}$ mice demonstrates a fragment 854bp in size that is consistent with the removal of TB, and slightly greater in size than the comparable product obtained from both liver and brain DNA extracts of the control mice. **b** Western blot image of total TFEB protein lysate in fed and fasted conditions from *Mc3r*$^{+/+}$, *Mc3r*$^{TB/TB,}$ and *Mc3r*$^{Hep/Hep}$ mice indicates similar total TFEB expression in all 3 mouse models in both fed and fasted states.

Supplementary Fig 3.

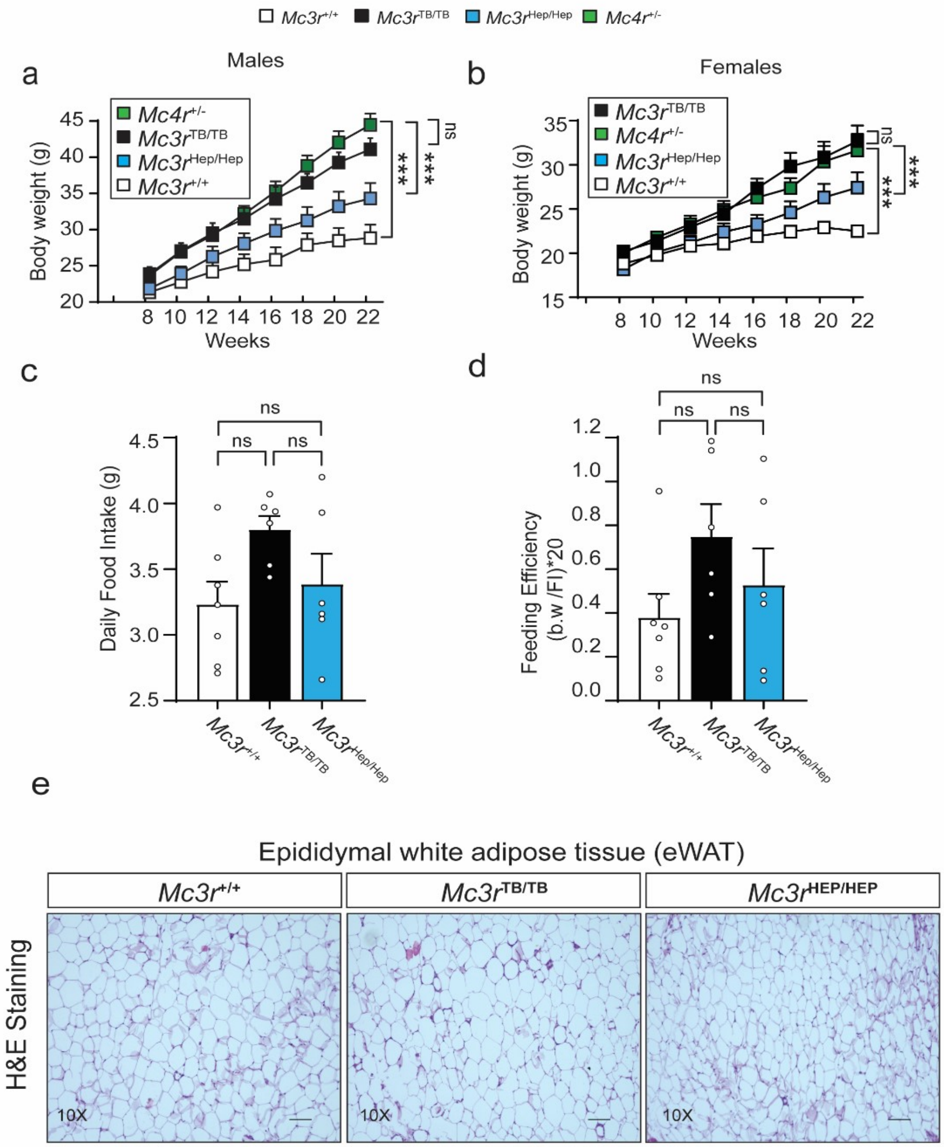

Supplementary Fig. 3. a Body weight measured from male *Mc3r*$^{+/+}$ (*n*=8), *Mc3r*$^{TB/TB}$ (*n*=9), *Mc4r*$^{+/-}$ (*n*=8) and *Mc3r*$^{Hep/Hep}$ (*n*=7) independent observations under chow diets. **b** Body weight measured from female *Mc3r*$^{+/+}$ (*n*=14), *Mc3r*$^{TB/TB}$ (*n*=8), *Mc4r*$^{+/-}$ (*n=8*), and *Mc3r*$^{Hep/Hep}$ (*n*=9) independent observations under chow diets. *Mc3r*$^{TB/TB}$ body weight compared to *Mc4r*$^{+/-}$ confirms *Mc3r* deficiency is a moderate form of obesity. **c** Daily Food Intake and **d** Feeding Efficiency from *Mc3r*$^{+/+}$, *Mc3r*$^{TB/TB}$ and *Mc3r*$^{Hep/Hep}$(*n*≥6 independent observations/genotype). **e** H&E staining for epididymal white adipose tissue also confirms partial reversal of the obesity phenotype when hepatic *Mc3r* is restored**.** Data are represented as mean±SEM. Groups were compared by two-way ANOVA followed by Tukey's HSD test (**a, b**), one-way ANOVA followed by Tukey's HSD test (**c, d**). Indicates values are significantly different * p<0.05; *** p<0.0001. Scale bar, 100 µm (**e**).

Supplementary Fig 4.

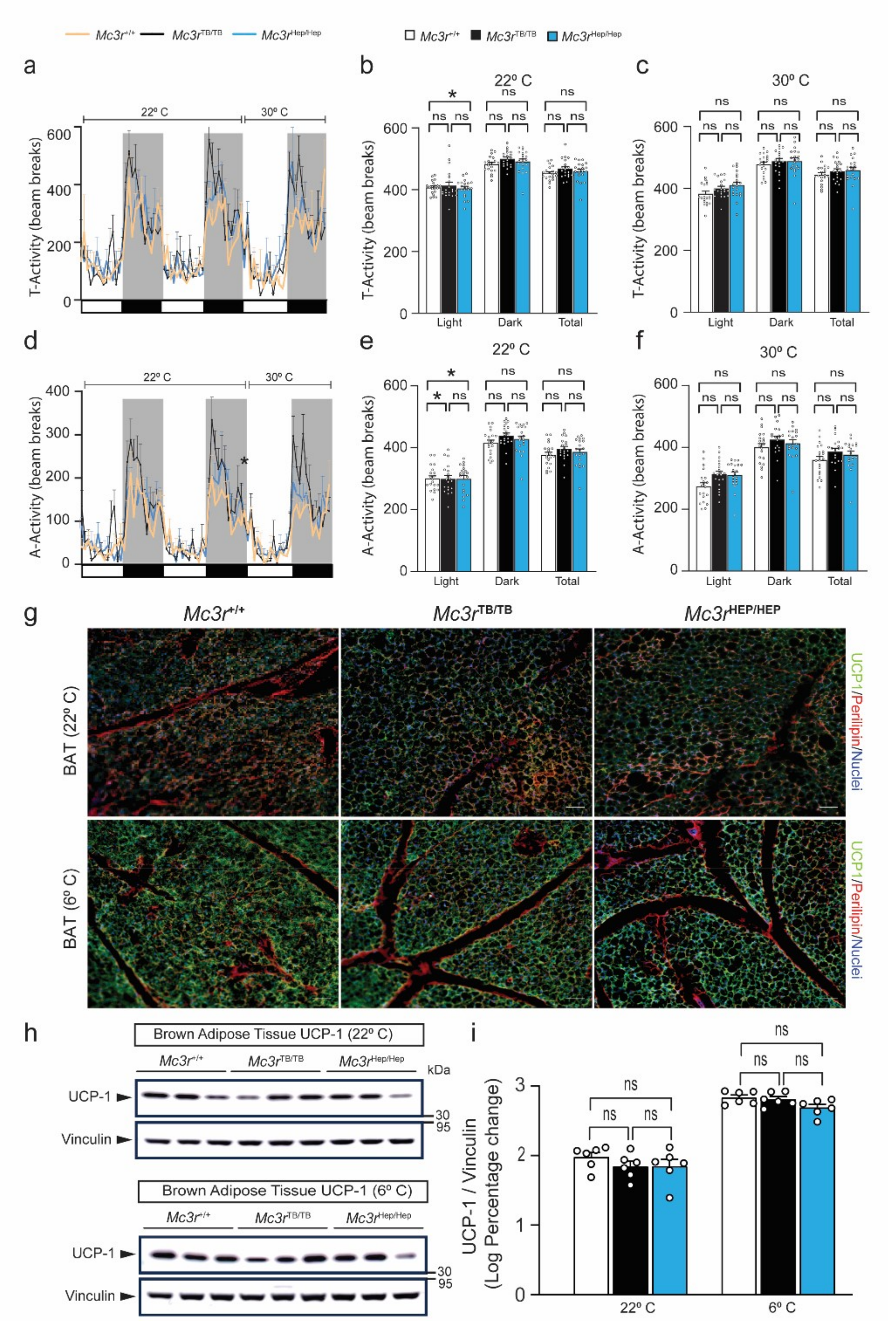

Supplementary Fig. 4. Locomotion activity and brown adipose tissue thermogenesis. **a** Total activity (T-activity) monitored for light and dark phase at ambient 22 °C and thermoneutral temperature 30°C from *Mc3r*$^{+/+}$, *Mc3r*$^{TB/TB}$ and *Mc3r*$^{Hep/Hep}$ (*n*=20) independent observations. **b** T-activity for light, dark, and total (24h) at 22°C. **c** T-activity for light, dark, and total (24h) at 30°C. **d** Ambulatory activity (A-Activity) monitored for light and dark phase at 22°C and 30°C. **e** A-activity for light, dark, and total (24h) at 22 °C. **f** A-activity for light, dark, and total (24h) at 30°C. Chow-fed, from *Mc3r*$^{+/+}$, *Mc3r*$^{TB/TB}$ and *Mc3r*$^{Hep/Hep}$ (*n*=20) independent observations mice 3-

4 months of age. **g** UCP1 immunofluorescence (IF) in brown adipose tissue (BAT) from *Mc3r*$^{+/+}$, *Mc3r*$^{TB/TB}$, and *Mc3r*^{HepHep}. Brown adipose tissue was stained for UCP-1 and DAPI from *Mc3r*$^{+/+}$, *Mc3r*$^{TB/TB,}$ and *Mc3r*$^{Hep/Hep}$ (*n*=3) independent observations. after 6 hours of cold (6°C) vs room temperature (22°C) exposure **h** UCP-1 protein expression for BAT after 6 hours of room temperature (22°C) and 6 hours of cold (6°C) from *Mc3r*$^{+/+}$, *Mc3r*$^{TB/TB}$, and *Mc3r*^{HepHep} **i** Quantification of UCP-1 normalized by Vinculin in (h) from *Mc3r*$^{+/+}$, *Mc3r*$^{TB/TB}$ and *Mc3r*$^{Hep/Hep}$ (*n*=6*/genotype)* independent observations. Data are represented as mean±SEM. Groups were compared by one-way ANOVA followed by Tukey's HSD test (**b, c, e, f, i**). * p<0.05. Scale bar, 50 μm (**g**).

Supplementary Fig 5.

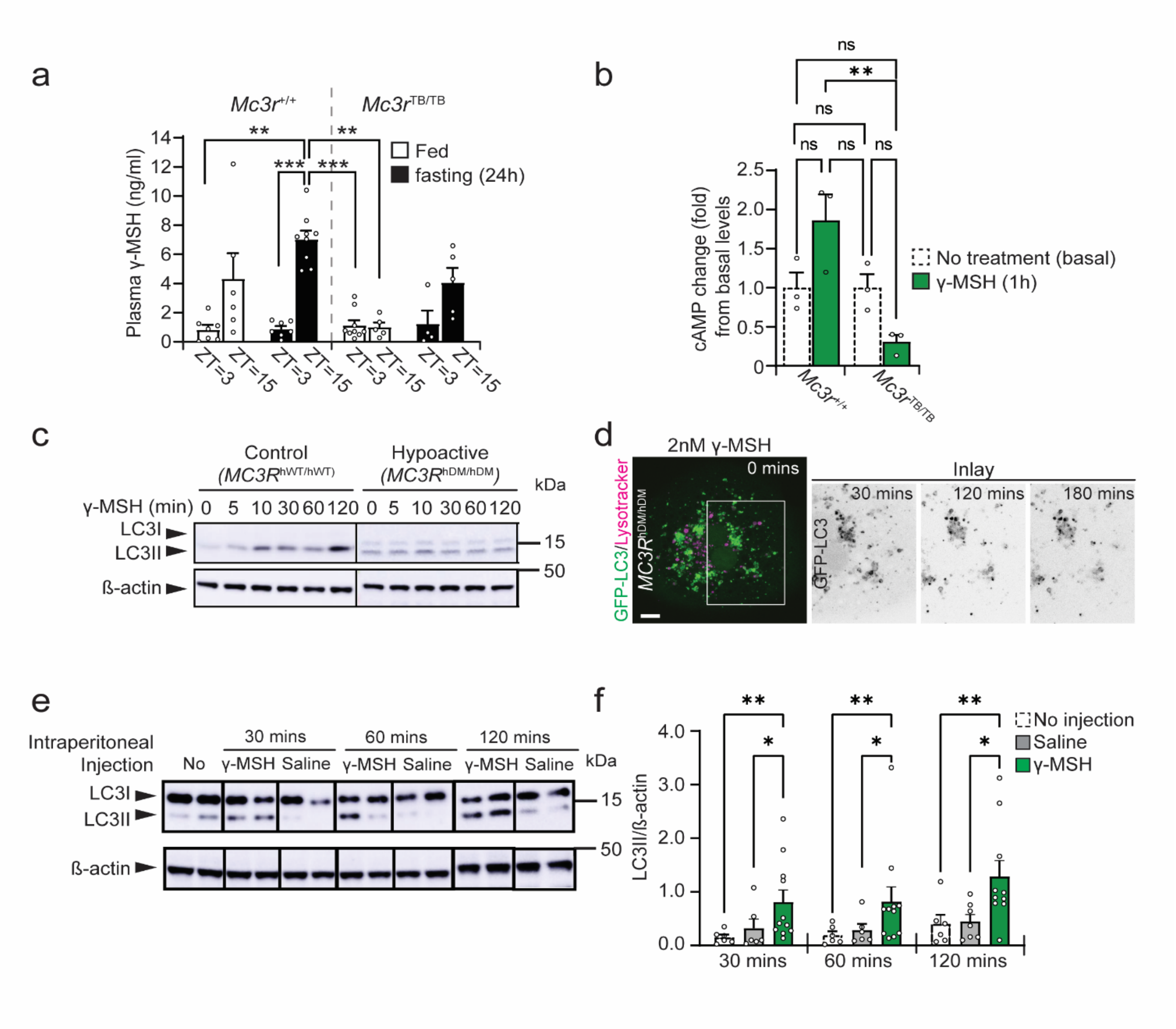

Supplementary Fig. 5. a Plasma γ-MSH concentrations were determined by ELISA from *$Mc3r^{+/+}$* and *$Mc3r^{TB/TB}$* mice under fed or starvation (24 hours) conditions from *$Mc3r^{+/+}$* (*n*=6 (fed) 9 (fasted)) at ZT15 and from *$Mc3r^{+/+}$* (*n*=7; fed and fasted each) at ZT3 and *$Mc3r^{TB/TB}$* liver (*n*=5; fed and fasted each) at ZT15, and from *$Mc3r^{TB/TB}$* liver (*n*=7 (fed) 4 (fasted)) at ZT3. **b** cAMP was determined by using ELISA method from primary hepatocytes isolated from *$Mc3r^{+/+}$* and *$Mc3r^{TB/TB}$* female mice (14 weeks of age) after 2nM D-Trp^8-γ-MSH (γ-MSH) treatment (*n=3*) independent observations. **c** LC3I and LC3II protein levels were measured after 2nM γ-MSH treatment in primary hepatocytes from *$Mc3r^{hWT/hWT}$ and $MC3R^{hDM/hDM}$* mice . **d** Representative time-lapse fluorescent images from *$MC3R^{hDM/hDM}$* transgenic hepatocytes carrying GFP-LC3 shown after 2nM γ-MSH treatment. **e** Representative western blot images of liver lysates from *$Mc3r^{+/+}$* mice after intraperitoneal γ-MSH at a dosage of 200 µg/kg body weight, or saline

injection that were prepared at indicated time points. **f** LC3II levels normalized to actin are shown (*n*=6 *for controls and n*=10 independent observations for γ-MSH). Groups were compared by two-way ANOVA followed by Tukey's HSD test (**a, f**), one-way ANOVA followed by Tukey's HSD test (**b**). * p<0.05; ** p<0.01; *** p<0.001. Scale bar, 10 µm (**d**).

Supplementary Fig 6.

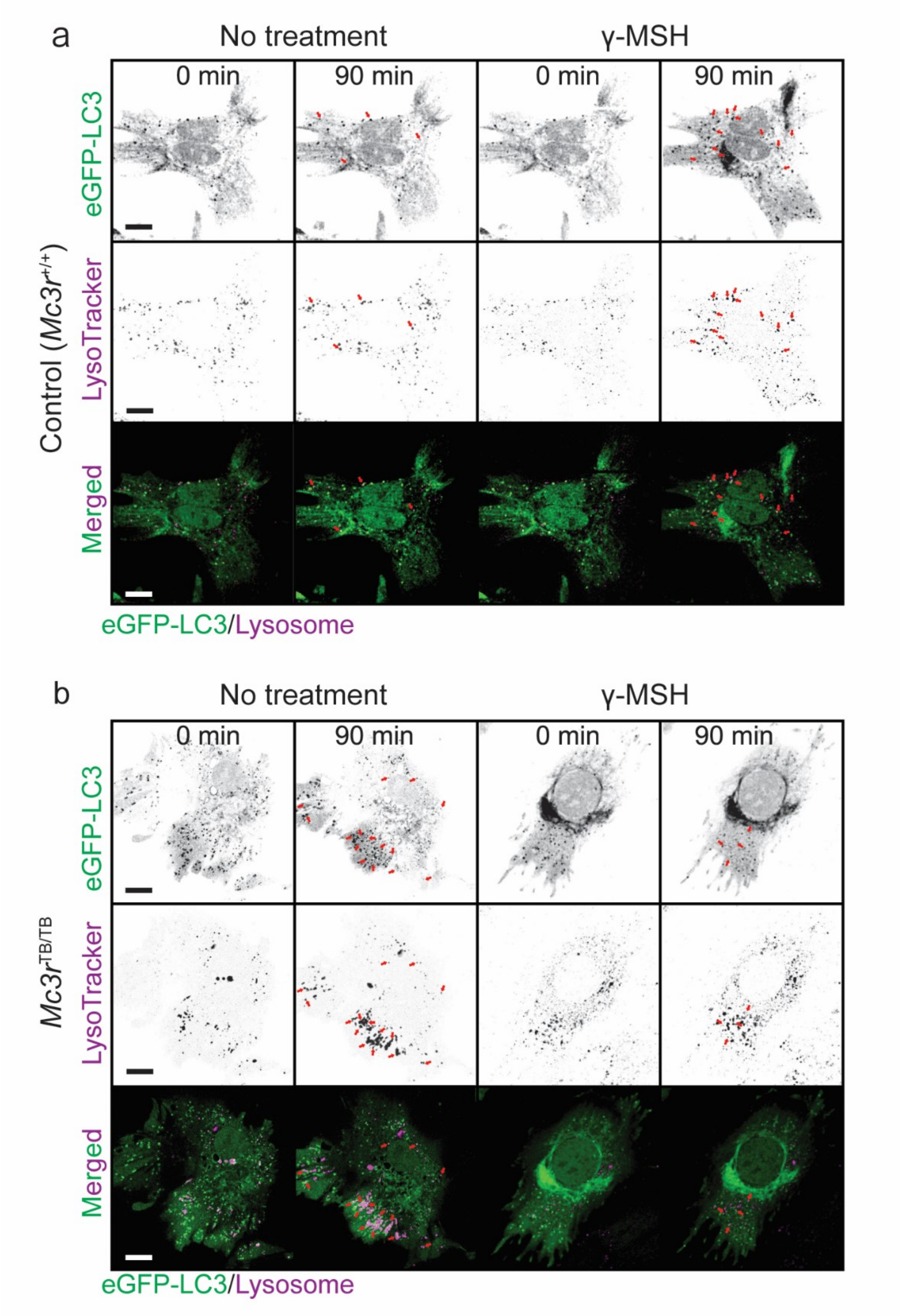

Supplementary Fig. 6. Representative time-lapse fluorescent images from transgenic hepatocytes carrying GFP-LC3 shown after no treatment or 2nM γ-MSH treatment of primary hepatocytes isolated from **a)** *Mc3r*$^{+/+}$ control and **b)** *Mc3r*$^{TB/TB}$ mice. Red arrowheads indicate GFP-LC3 autophagosome structures. Scale bar 10 μm.

Supplementary Fig 7.

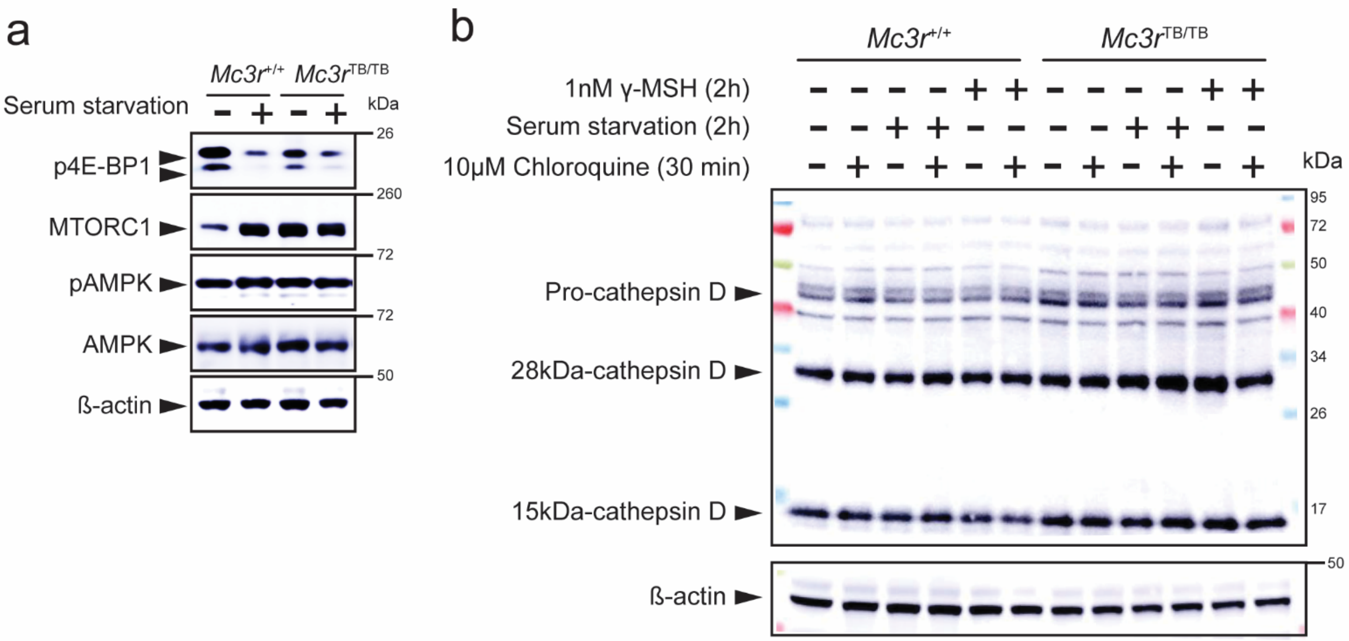

Supplementary Fig. 7. a Representative western blot images of primary hepatocytes from *Mc3r*$^{+/+}$ mice after serum starvation for 1 hour are shown. **b** Representative western blot images from *Mc3r*$^{+/+}$ or *Mc3r*$^{TB/TB}$ hepatocytes are shown after γ-MSH treatment, serum starvation, and/or chloroquine treatment. Anti-Cathepsin D antibody was used to monitor the lysosomal maturation process.

Supplementary Table 1.

Energy intake and energy expenditure-related parameters adjusted for body composition in chow-fed condition.									
Group	Energy Intake kcal adj. for Body Weight	Energy Intake kcal adj. for Lean & Fat mass	Energy Intake kcal adj. for Lean mass	TEE Kcal/mouse/h adj. for Body Weight	TEE Kcal/mouse/h adj. for Lean & Fat mass	TEE Kcal/mouse/h adj. for Lean mass	VO_2 (mL/mouse/h) adj. for Body Weight	VO_2 (mL/mouse/h) adj. for Lean & Fat mass	VO_2 (mL/mouse/h) adj. for Lean mass
**A.** *Mc3r*$^{+/+}$ (22°C)	10.94±0.456	10.94±0.455	10.83±0.469	0.408±0.007	0.409±0.007	0.402±0.008	83.36±1.62	83.48±1.71	82.47±1.82
**B.** *Mc3r*$^{TB/TB}$ (22°C)	8.67±0.457	8.67±0.455	8.76±0.460	0.387±0.007	0.386±0.007	0.390±0.008	77.18±1.62	76.92±1.71	77.43±1.78
**C.** *Mc3r*$^{HEP/HEP}$ (22°C)	8.68±0.455	8.68±0.455	8.71±0.454	0.401±0.007	0.401±0.007	0.404±0.007	79.82±1.62	79.90±1.80	80.40±1.76
p-value (A-B)	*0.001*	*0.001*	*0.004*	*0.032*	*0.032*	*0.308*	*0.009*	*0.009*	*0.058*
p-value (A-C)	*0.001*	*0.009*	*0.002*	*0.435*	*0.466*	*0.829*	*0.128*	*0.440*	*0.423*
p-value (B-C)	*0.996*	*0.990*	*0.941*	*0.162*	*0.150*	*0.199*	*0.244*	*0.222*	*0.236*
**A.** *Mc3r*$^{+/+}$ (30°C)	8.99±0.526	8.99±0.412	8.77±0.554	0.285±0.005	0.286±0.005	0.282±0.005	58.34±1.20	58.42±1.24	57.80±1.29
**B.** *Mc3r*$^{TB/TB}$ (30°C)	8.24±0.526	8.22±0.412	8.33±0.543	0.262±0.005	0.262±0.005	0.264±0.005	52.53±1.20	52.40±1.24	52.68±1.26
**C.** *Mc3r*$^{HEP/HEP}$ (30°C)	8.64±0.526	8.65±0.412	8.76±0.536	0.275±0.005	0.276±0.005	0.277±0.005	54.81±1.19	54.87±1.23	54.18±1.24
p-value (A-B)	*0.318*	*0.298*	*0.584*	*0.002*	*0.002*	*0.029*	*0.001*	*0.001*	*0.007*
p-value (A-C)	*0.639*	*0.640*	*0.997*	*0.163*	*0.180*	*0.583*	*0.041*	*0.047*	*0.152*
p-value (B-C)	*0.592*	*0.562*	*0.568*	*0.071*	*0.067*	*0.082*	*0.183*	*0.165*	*0.159*

Supplementary Table 1: Energy Intake (kcal), Total energy expenditure (TEE: Kcal/mouse/h) and oxygen consumption rate (VO_2: mL/mouse/h) of chow-fed (*n*=20/group) 10-male and 10-female mice at 22 °C and 30 °C were measured over a 24-hour period by indirect calorimetry. Total energy was calculated using the 3.05 kcal/g chow diet research diet. The average 24-hour energy expenditure was adjusted for multiple factors by analysis of covariance (ANCOVA). The adjusted Energy intake, TEE, and VO_2 values shown in the table are estimated marginal means from the ANCOVA adjusted for body weight, lean & fat mass, or lean mass. Respiratory Exchange Ratio (RER) is not shown because it is a ratio calculated for each mouse and is therefore not generally adjusted for body composition. Estimated marginal means are shown. *Mean ± SEM. *p*-value (A-B; *Mc3r*$^{+/+}$ vs. *Mc3r*$^{TB/TB}$), *p*-value (A-C; *Mc3r*$^{+/+}$ vs. *Mc3r*$^{Hep/Hep}$), *p*-value (B-C; *Mc3r*$^{TB/TB}$ vs. *Mc3r*$^{Hep/Hep}$).

Supplementary Table 2.

Material and Reagents:	SOURCE	IDENTIFIER
Antibodies		
Rabbit monoclonal anti-LC3A/B (D3U4C)	Cell Signaling Technology	12741
Rabbit polyclonal anti- SQSTM1/p62	Cell Signaling Technology	5114
TFEB	Bethyl	A303-673A
Histone H3 (1B1B2)	Cell Signaling Technology	14269
Phospho-4E-BP1	Cell Signaling Technology	2855
mTOR	Cell Signaling Technology	2972
Phospho-AMPKα (D79.5E)	Cell Signaling Technology	4188
AMPKα (D5A2)	Cell Signaling Technology	5831
Mouse monoclonal anti-β Actin	Abcam	AB20272
Anti-UCP1	Abcam	AB155117
Anti-Vinculin	Abcam	AB129002
Anti-Cathepsin D	Abcam	AB302650
Anti-Perilipin	LSBio	Ls-C193442
Chemicals, Peptides, and Recombinant Proteins		
[D-Trp^{8}]-γ-MSH	Phoenix Pharmaceuticals	043-10
NDP-MSH-42	C-H-L, University of Minnesota	KAF4098-32
Oil Red O	Sigma	O0625
Chloroquine	Cell Signaling Technology	14774S
Rapamycin	Cell Signaling Technology	9904S
DAPI	Thermo Fisher Scientific	D1306
LysoTracker Red	Invitrogen	L7528
Insulin	Sigma	I6634
Glucose	Sigma	G7021
Critical Commercial Assays		
MSH, gamma 2 (Human, Rat, Mouse)	Phoenix Pharmaceuticals Inc	EK-043-16 &RK-Sepcol-1
Mito Stress Test	Agilent	103015-100
Direct cAMP ELISA kit	ENZO Life sciences	ADI-900-066
Rat Insulin ELISA kit	Crystal Chem	90010
Mouse serum NEFA Kit	Fujifilm Wako	991-34891
Glycerol assay kit	Sigma-Aldrich	MAK117
LabAssay™ Triglyceride	Fujifilm Wako	291-94501
LabAssay (TM) Cholesterol	Fujifilm Wako	635-50981
Hematoxylin and eosin staining kit	Tissue TEK	SKU#6190
Oligonucleotides		
Primers for GABARAP	IDT	NM_007278
Primers for SQSTM1	IDT	NM_001142298
Primers for MCOLN1	IDT	NM_020533
Primers for NEU1	IDT	NM_000434
Primers for CD36	IDT	Mm.PT.58.7548967
Primers for Cidec	IDT	Mm.PT.58.6462335
Primers for PGC-1a	IDT	Mm.PT.58.16192665
Primers for FAS	IDT	Mm.PT.58.41299055
Primers for HSL	IDT	Mm.PT.58.6342082
Primers for β-Actin	IDT	Mm.PT.39a.22214843.g
Primers for Ywhaz	IDT	Mm.PT.39a.22214831

Supplementary Table 2: List of materials and reagents, with the companies from which they came, and catalog numbers.

Supplementary Table 3.

Resources: Experimental Models	Source	Identifier
Experimental Models: Cells		
Primary cultured mouse hepatocytes	Mouse	
Experimental Models: Organisms/Mice Strains		
Mouse: C57B/L6j	The Jackson Laboratory	Stock No: 000664
Mouse: MC3R$^{TB/TB}$, B6(Cg)-*Mc3r*^{tm1Butl}/J	The Jackson Laboratory	Stock No: 017866
Mouse: GFP-LC3	Gift from Dr. Noboru Mizushima (RIKEN BRC)	RBRC No: RBRC00806
Mouse: MC3R$^{hWT/hWT}$: Mc3r$^{tm1.1(MC3R)Jayk}$	This paper, Lee *et al.* 2016	MGI:6382605
Mouse: MC3R$^{hDM/hDM}$: Mc3r$^{tm2.1(MC3R^*)Jayk}$	This paper, Lee *et al.* 2016	MGI:6382576
Mouse: MC3R$^{hWT/hWT}$/GFP-LC3	This paper	Breeding Colony
Mouse: MC3R$^{hDM/hDM}$/GFP-LC3	This paper	Breeding Colony
Mouse: MC4R$^{+/-}$ B6;129S4-*Mc4r*^{tm1Lowl}/J	The Jackson Laboratory	Stock No: 032518
Mouse: B6.Cg-*Speer6-ps1*$^{Tg(Alb\text{-}cre)21Mgn}$/J	The Jackson Laboratory	Stock No: 003574
Mouse: MC3R$^{Hep/Hep}$	This paper	Breeding Colony
Rodent Diet		
Open formula rat and mouse ration (NIH-07)	Lab Diet Arden Hills, MN	5018

Supplementary Table 3: Experimental models employed, with source of mice and stock numbers where available.

Uncropped Images for Figures

Fig 2d

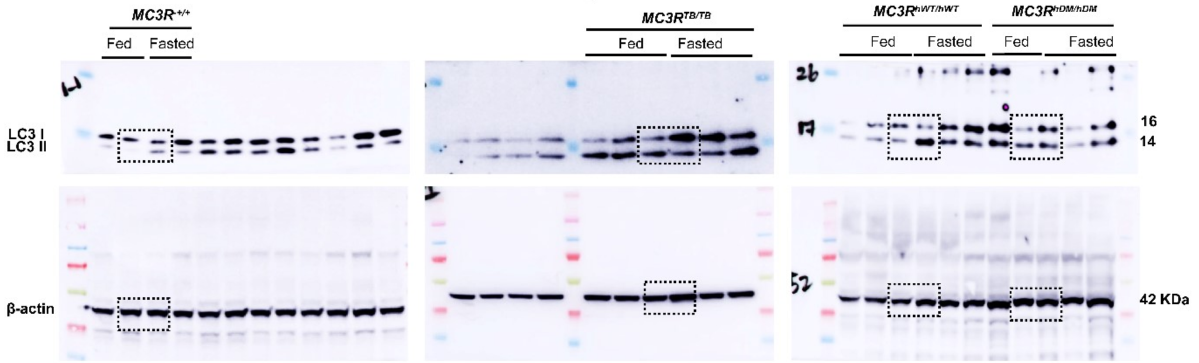

Fig 2f

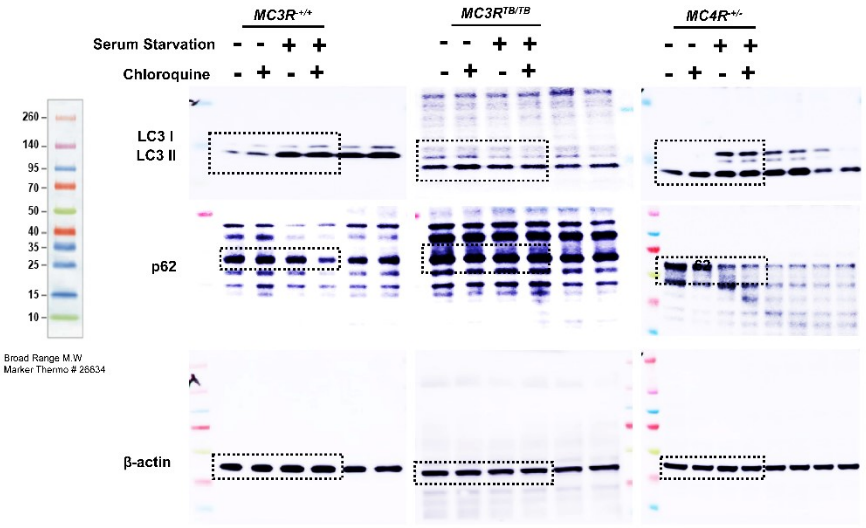

Fig 6k

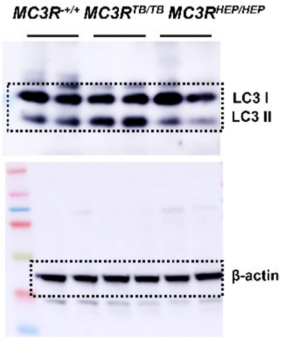

Fig 7c

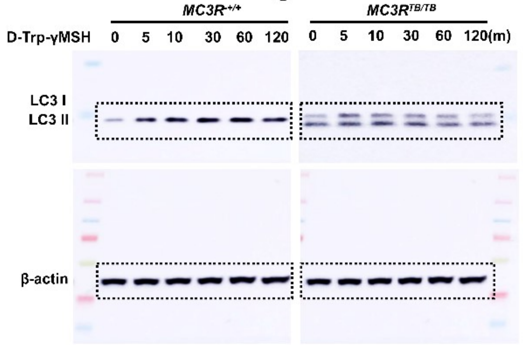

Fig 7f

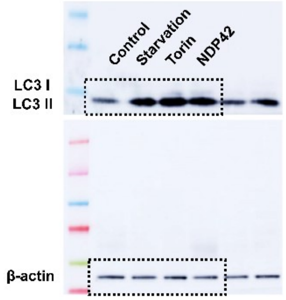

Fig 7h

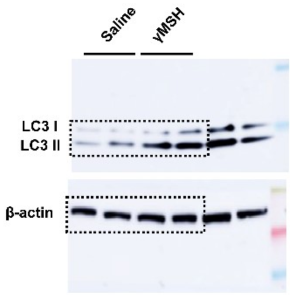

Fig 7j

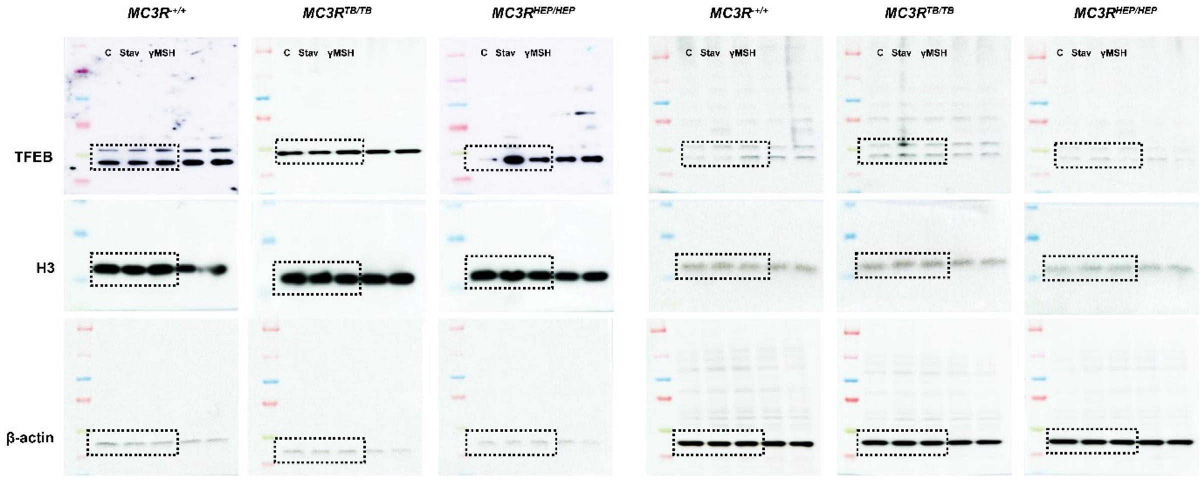

Uncropped Images for Supplementary Figures

S.Fig 1c

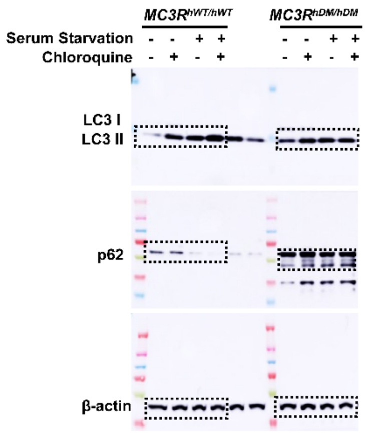

S.Fig 2b

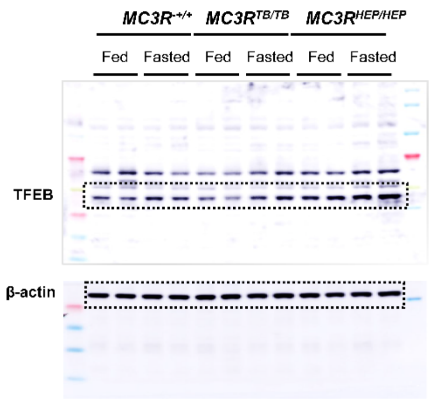

S.Fig 4h

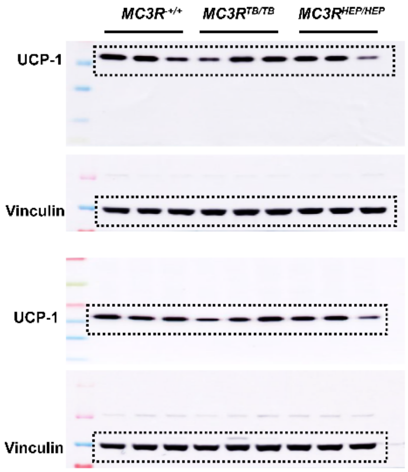

S.Fig 5c

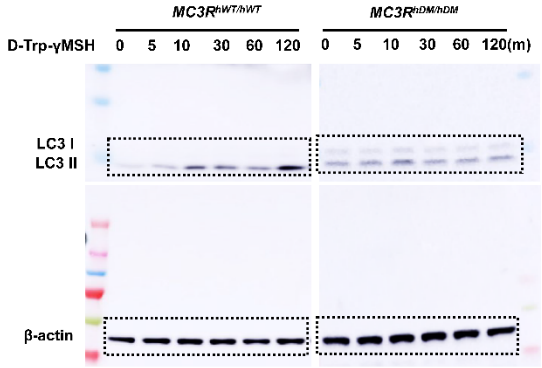

S.Fig 5e

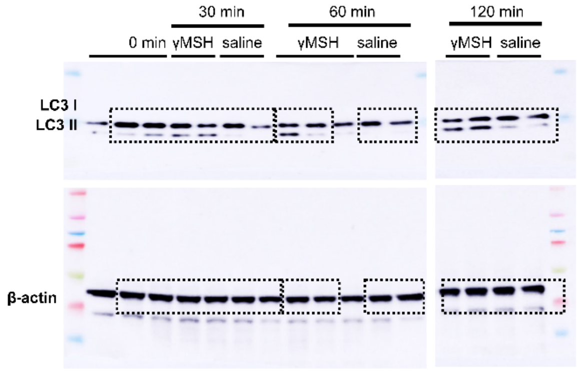

S.Fig 7a

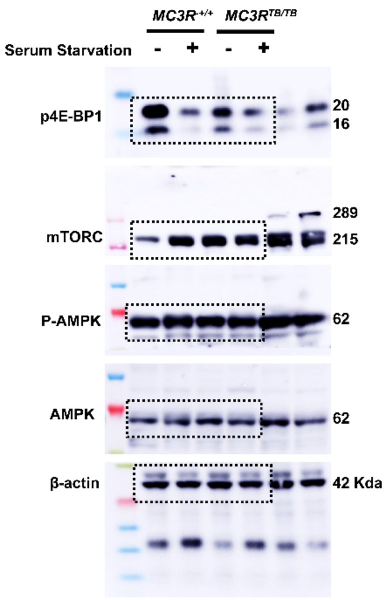

S.Fig 7b

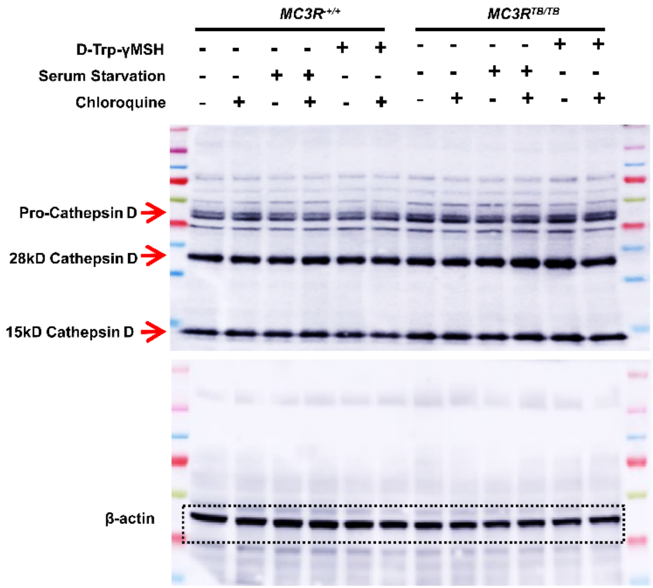

Supplement: Supplementary file 1 — Supplementary Information [file 41467_2025_56936_MOESM1_ESM.pdf]
